# Genome-wide association studies for yield component traits in a macadamia breeding population

**DOI:** 10.1186/s12864-020-6575-3

**Published:** 2020-03-04

**Authors:** Katie O’Connor, Ben Hayes, Craig Hardner, Catherine Nock, Abdul Baten, Mobashwer Alam, Robert Henry, Bruce Topp

**Affiliations:** 1 Queensland Department of Agriculture and Fisheries, Maroochy Research Facility, Nambour, Qld Australia; 2 Queensland Alliance for Agriculture and Food Innovation, University of Queensland, St Lucia, Qld Australia; 3 Southern Cross Plant Science, Southern Cross University, Lismore, NSW Australia; 4 AgResearch, Grasslands Research Centre, Palmerston North, New Zealand

**Keywords:** Horticulture, Plant breeding, Progeny, Genomics, Marker-assisted selection, Nut

## Abstract

**Background:**

Breeding for new macadamia cultivars with high nut yield is expensive in terms of time, labour and cost. Most trees set nuts after four to five years, and candidate varieties for breeding are evaluated for at least eight years for various traits. Genome-wide association studies (GWAS) are promising methods to reduce evaluation and selection cycles by identifying genetic markers linked with key traits, potentially enabling early selection through marker-assisted selection. This study used 295 progeny from 32 full-sib families and 29 parents (18 phenotyped) which were planted across four sites, with each tree genotyped for 4113 SNPs. ASReml-R was used to perform association analyses with linear mixed models including a genomic relationship matrix to account for population structure. Traits investigated were: nut weight (NW), kernel weight (KW), kernel recovery (KR), percentage of whole kernels (WK), tree trunk circumference (TC), percentage of racemes that survived from flowering through to nut set, and number of nuts per raceme.

**Results:**

Seven SNPs were significantly associated with NW (at a genome-wide false discovery rate of < 0.05), and four with WK. Multiple regression, as well as mapping of markers to genome assembly scaffolds suggested that some SNPs were detecting the same QTL. There were 44 significant SNPs identified for TC although multiple regression suggested detection of 16 separate QTLs.

**Conclusions:**

These findings have important implications for macadamia breeding, and highlight the difficulties of heterozygous populations with rapid LD decay. By coupling validated marker-trait associations detected through GWAS with MAS, genetic gain could be increased by reducing the selection time for economically important nut characteristics. Genomic selection may be a more appropriate method to predict complex traits like tree size and yield.

## Background

Macadamia is a large nut tree native to the coastal rainforests of southern Queensland and northern New South Wales, Australia. *Macadamia integrifolia* Maiden & Betche, *M. tetraphylla* L.A.S. Johnson and their hybrids have high-quality edible kernels, and are the first indigenous Australian food species to be commercialised internationally. The industry is largely based on cultivars developed in Hawaii in the late nineteenth century [1]. Current production is dominated by Australia, South Africa and Hawaii, and is expanding in China, Kenya and other countries around the world [2]. A major focus in breeding new macadamia varieties is increasing nut-in-shell yield per tree. However, the heritability of yield is low (H^2^ ≈ 0.12), largely influenced by environment, and, as such, difficult to select [3]. To date, conventional phenotype- and pedigree-based selection has been employed to improve yield of commercial varieties. Long juvenile periods, large tree sizes and labour involved in phenotyping over continuous years to identify elite candidate cultivars mean that fruit and nut trees may benefit from genomic approaches to reduce selection cycles and increase genetic gain [4].

The use of genomics in plant breeding is expanding [4–6], including employing genome-wide association studies to identify molecular markers associated with important traits, and genomic selection for complex traits. A common approach is using genome-wide association studies (GWAS): each marker (typically single nucleotide polymorphism, SNP) is tested individually to detect evidence of marker-trait associations [4]. This method relies on linkage disequilibrium (LD) between markers and causal polymorphisms [4]. To avoid spurious genotype-phenotype association due to population structure and family structures, linear mixed models, fitting individuals as random effects to account for relatedness, are widely used. As the realised kinship estimated from genetic markers is more accurate than recorded pedigree, fitting genomic relationships in the model can reduce false positives of putative large-effect QTLs [7, 8]. QTLs identified through GWAS can be followed by marker-assisted selection (MAS) if a reasonable proportion of trait genetic variation is explained by the significant markers. In MAS, candidates are screened for target markers, their phenotypes are predicted based on allelic states, and selections can be made based on these predictions [9, 10].

Several fruit and nut crops have employed GWAS to identify markers associated with key traits [11–18]. Furthermore, by mapping significant markers to reference genomes, the location of markers can be determined in order to investigate candidate genes, although this is not necessary for MAS. GWAS coupled with MAS at these specific loci is a feasible option for improving yield component traits in macadamia [19]; hence, we aim to investigate this option in the Australian macadamia breeding program.

Target traits for GWAS and potential MAS in macadamia include commercially important traits, such as nut and flowering characteristics, as well as tree size. Nuts consist of an inner edible kernel, with two cotyledons, which is enclosed by a hard shell (testa) and outer husk (pericarp) [1, 20]. Nut weight (NW), kernel weight (KW), and kernel recovery (KR) are commercially important yield component traits. For NW and KW, the industry favours intermediate optimums (6.5–7.5 g and 2–3 g, respectively) due to issues involved in handling, cracking, processing, and roasting smaller and larger nuts [1]. The selection goal for KR, which is the proportion of kernel to nut-in-shell (KW/NW), may not be completely clear. Whilst high (> 37%) KR attracts a premium price per kilogram [21], very thin shells can be prone to pest and disease damage [1]. Whole kernels (WK) are those that have not split along the interface separating the two cotyledons during cracking [22]; this trait can influence kernel price as some products and markets prefer whole kernels [1, 23].

Macadamia trees can produce about 2500 pendant racemes 6–30 cm long, each with an inflorescence of 100–300 florets [24, 25]. It has been estimated that less than 1% of florets produce viable nuts [26]. This estimate, therefore, indicates that many racemes and florets fail, likely due to a variety of reasons, and resource allocation may be a factor. As such, the percentage of racemes that survive from flowering through to nut set (RSN) could indicate a genotype’s reproduction success and energy investments, in terms of resource allocation for flowering versus nut retention [27, 28]. Reduced tree size is also an important selection trait to increase planting density and subsequent yield per hectare [29, 30]. Trunk circumference (TC) or trunk cross-sectional area can be used as an estimate of tree size in macadamia [30].

O’Connor [31] investigated heritability and correlations of yield and yield component traits measured on mature progeny. Several commercially important traits, as well as flowering and nut set characteristics that were moderately or highly correlated with yield are the focus of this study. It is hypothesised that marker-trait associations will be detected for these key traits using GWAS, and upon validation could be combined with MAS to improve breeding efforts and increase genetic gain in macadamia. The current study builds on work previously published in a preliminary study [32] on the same population of trees. In that preliminary study, O’Connor et al. [32] found SNP markers associated with three nut characteristics (NW, KW and KR) measured on trees at the ages of 7–9 years (in 2010). In comparison, the current study uses a different set of SNP markers imputed with high accuracy, and performs GWAS on yield component traits measured on the same trees at a mature age (aged 14–17 years, in 2016–2018). The aims of this study were to: (i) perform GWAS to identify markers significantly associated with yield component traits, and (ii) determine the location of significant markers on genome scaffolds.

## Results

### Component traits

**Fig. 1:**
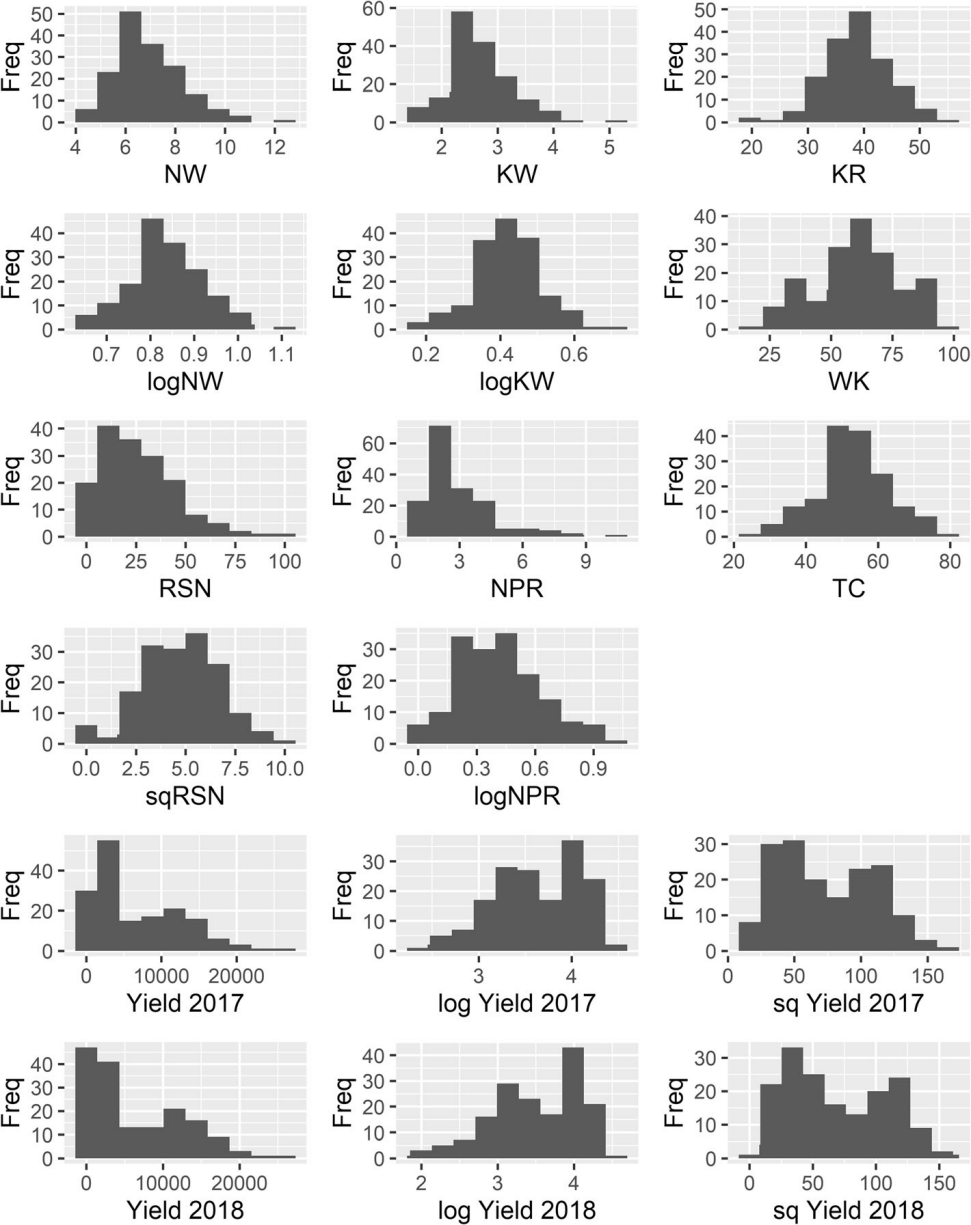
Distribution of phenotypes across all individuals for yield component traits. Freq, frequency; NW, nut weight; KW, kernel weight; KR, kernel recovery; WK, percentage of whole kernels; RSN, percentage of racemes that set nuts; NPR, number of nuts per raceme; TC, trunk circumference. Log-transformed (log_10_(x)) NW, KW and NPR, and square root transformed (sq) RSN distributions are also shown, as well as both forms of transformation for yield in 2017 and 2018

Raw (untransformed) phenotypes for KR, WK and TC were normally distributed (Fig. 1). Log-transformed (log_10_(x)) observations for NW, KW and NPR, as well as square root transformed observations for RSN appeared more normally distributed than raw observations (Fig. 1). Yield (2017 and 2018) was not normally distributed, and neither log (log_10_(x), ln) nor square root transformations led to more normally distributed data, even for individual sites. This indicates that GWAS is not appropriate for yield, and association analysis was not performed for this trait.

Phenotypes ranged from 4.34 to 12.31 g for NW, 1.46 to 5.01 g for KW. As a derivative of these two traits, KR ranged from 20.2 to 55.6% (Table 1). Moderate to high correlations (*p* < 0.01) were observed between young and mature phenotypes for NW, KW and KR (0.56, 0.66 and 0.73; Table 1). For three genotypes, including cultivar ‘Yonik’, there were no broken kernels (100% WK) in the sample, whilst one tree possessed a very low WK (15%). Most small trees (small TC) were observed at site EG, with the lowest TC at 14 cm. Conversely, trees with large TC were observed at the AL and HP sites, with a maximum TC of 78 cm at site HP. An entire range of phenotypes was observed for RSN, from 0 to 100%, with a mean of 25%. Mean NPR was 2.6 and ranged from 1 to 10.4 (Table 1).

**Table 1:** Summary of raw (untransformed) phenotypes for each trait analysed in GWAS

Trait	Min	Max	Mean	SD	r_p_
NW (g)	4.34	12.31	7.09	1.34	0.56
KW (g)	1.46	5.01	2.73	0.55	0.66
KR (%)	20.2	55.6	38.7	5.4	0.73
WK (%)	15	100	64	17	–
TC (cm)	14	78	51	12	–
RSN (%)	0	100	25	18	–
NPR	1	10.4	2.6	1.4	–

*SD* standard deviation, *r*_*p*_, Pearson’s correlation of current data with raw phenotypes for young trees from O’Connor et al. [32]

### Trait-specific models and heritability

For all traits except RSN, the most parsimonious model included site as a significant fixed effect, whilst block within site was also significant for NW and TC (Table 2). Tree type was included in the WK model, with a significance level of *p* = 0.063. The G x E term was included as a random effect for NW and NPR (Table 2). Narrow-sense genomic heritability varied across traits, from 0.08 for RSN to 0.74 for KR (Table 2). TC and NW were moderately heritable (0.45 and 0.53, respectively).

**Table 2:** Significance values of fixed and random terms included in association analysis model for each trait

Trait	Site	Block	Tree Type	G x E	h^2^
NW^b^	0.0014	0.0025		^a^	0.53
KW^b^	1.682e-13				0.37
KR	1.916e-09				0.74
WK	8.852e-05		0.063		0.24
TC	< 2.2e-16	0.0043			0.45
RSN^b^					0.08
NPR^b^	3.017e-08			^a^	0.09

Type, seedling progeny or grafted parents; G x E, genotype by environment (site) interaction; h^2^, narrow-sense heritability. Non-significant *p*-values (*p* > 0.05) are not shown and were not included in models, except for Type for WK. ^a^ indicates G x E model was significantly better fitting than model without G x E term, as determined using log-likelihood ratio test. h^2^ estimated from the best-fitting model with the GRM fitted. ^b^ indicates data were transformed

### Genome-wide associations

**Fig. 2:**
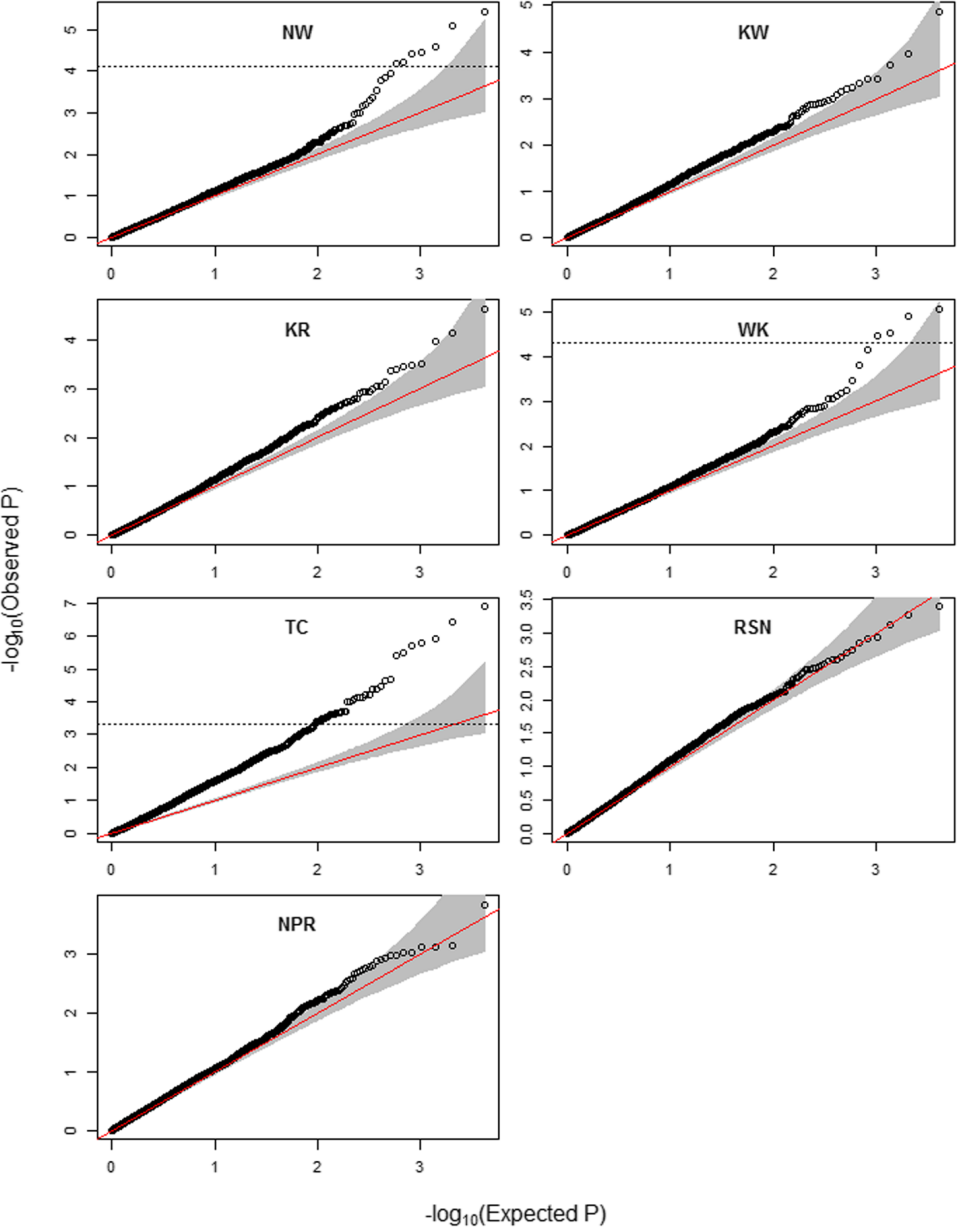
QQ plots showing expected significance levels against observed significance for yield component traits. Each circle represents one of 4113 SNP markers. Red diagonal lines indicate the null hypothesis, where observed and expected *p*-values would sit if there were no associations. Dashed horizontal lines indicate FDR = 0.05, SNP markers above which were deemed significantly associated with the trait; if no dashed horizontal line is present then no SNPs exceeded the FDR threshold. Shaded area indicates 95% confidence interval

The GRM appeared to have effectively accounted for population structure in all traits except for TC, as no more associations than expected by chance were observed at low levels of significance in the QQ plots (Fig. 2) [33]. GWAS identified seven SNP markers significantly (FDR < 0.05) associated with NW, four with WK, and 44 with TC (Fig. 2; Table 3). For both KW and KR, no markers exceeded the FDR threshold; however, there was one marker of interest in both traits that were further investigated. There were no markers significantly associated with RSN or NPR.

**Table 3:** Summary of significant SNPs associated with yield component traits identified in GWAS

Trait	SNP	Scaffold^a^	Position (bp)	Alleles	MAF	p	pMR	BLUE
NW^c^	s2204	scaffold926|size239084	212,122	A/G	0.027	3.68E-06	4.46e-06	0.084
	s4163	scaffold285|size451335	314,657	C/T	0.027	8.03E-06	NS	
	s1434	scaffold_177|size983250	804,678	T/C	0.019	2.65E-05	NS	
	s1643	scaffold44|size832018	129,241	A/C	0.021	3.46E-05	NS	
	s1121	scaffold653|size305054	6573	A/G	0.021	3.82E-05	NS	
	s5182	–	–	A/T	0.035	6.29E-05	NS	
	s2256	scaffold710|size289053	142,496	G/T	0.026	6.45E-05	NS	
KW^c^	s3540^b^	∫	∫	G/A	0.482	1.34E-05		
KR	s1707^b^	scaffold_72|size1196525	587,142	C/T	0.061	2.37E-05		
WK	s0201	scaffold213|size509421	186,179	G/A	0.093	8.81E-06	1.11E-06	4.608
	s3239	scaffold361|size1112638	1,087,419	G/C	0.037	3.39E-05	2.45E-04	−10.359
	s1917	–	–	A/G	0.163	1.23E-05	NS	
	s2607	–	–	T/C	0.177	2.91E-05	NS	
TC	s3169	scaffold146|size572432	176,797	T/C	0.230	1.29E-07	1.13E-07	−1.343
	s1885	∫	∫	C/T	0.319	8.57E-05	4.85E-05	−1.706
	s2320	scaffold81|size707423	173,614	C/A	0.083	1.02E-04	3.90E-05	4.088
	s3332	scaffold1221|size537814	497,497	T/C	0.285	1.97E-06	3.98E-04	2.167
	s1208	∫	∫	C/T	0.179	3.14E-04	6.96E-04	−2.383
	s3291	∫	∫	G/T	0.267	4.09E-05	7.52E-04	0.540
	s4709	∫	∫	G/A	0.106	4.74E-04	2.62E-03	−11.946
	s3311	–	–	A/C	0.043	3.90E-04	3.81E-03	−4.442
	s3828	∫	∫	G/A	0.093	4.03E-04	4.47E-03	−2.009
	s2230	scaffold_88	424,720	G/T	0.884	2.03E-04	6.15E-03	−2.360

Only the ten most significant markers for TC are shown. MAF, minor allele frequency of the marker; p, significance of association; pMR, significance of association as determined by multiple regression with significant SNPs as fixed effects; BLUE, best linear unbiased estimator (fixed effect) of SNP, additive effect of allele on the trait; NS, not significant. - indicates marker was not mapped to scaffolds. ∫ indicates marker was mapped to multiple scaffolds. ^a^ Scaffold in v2 genome assembly. ^b^ Did not pass FDR = 0.05 threshold. ^c^ indicates data were transformed

After multiple regression, where significant SNPs were treated as fixed effects, some markers were no longer significantly associated with some traits. Only SNP s2204 remained significantly associated with NW, whilst for WK, the two mapped markers (mapped to different scaffolds) and another marker remained significant, but the unmapped SNP s2607 was redundant. The number of SNPs significantly associated with TC decreased to 16 after multiple regression analysis.

Fifty-two of the 57 (91%) significant SNPs across the traits were mapped to scaffolds of the v2 macadamia genome assembly (Table 3). Some markers mapped to multiple scaffolds, for example, s3710 was located on 51 different scaffolds. Most scaffolds only had one SNP mapped, though six scaffolds had two SNPs mapped each. Almost 50% allele frequency was observed for two markers (s3540 for KW, and s3616 for TC; Table 3). The BLUEs estimated for the significant markers from the multiple regression model ranged from - 10.359 to 4.608 for WK, and - 11.946 to 4.088 for TC (Table 3).

**Fig. 3:**
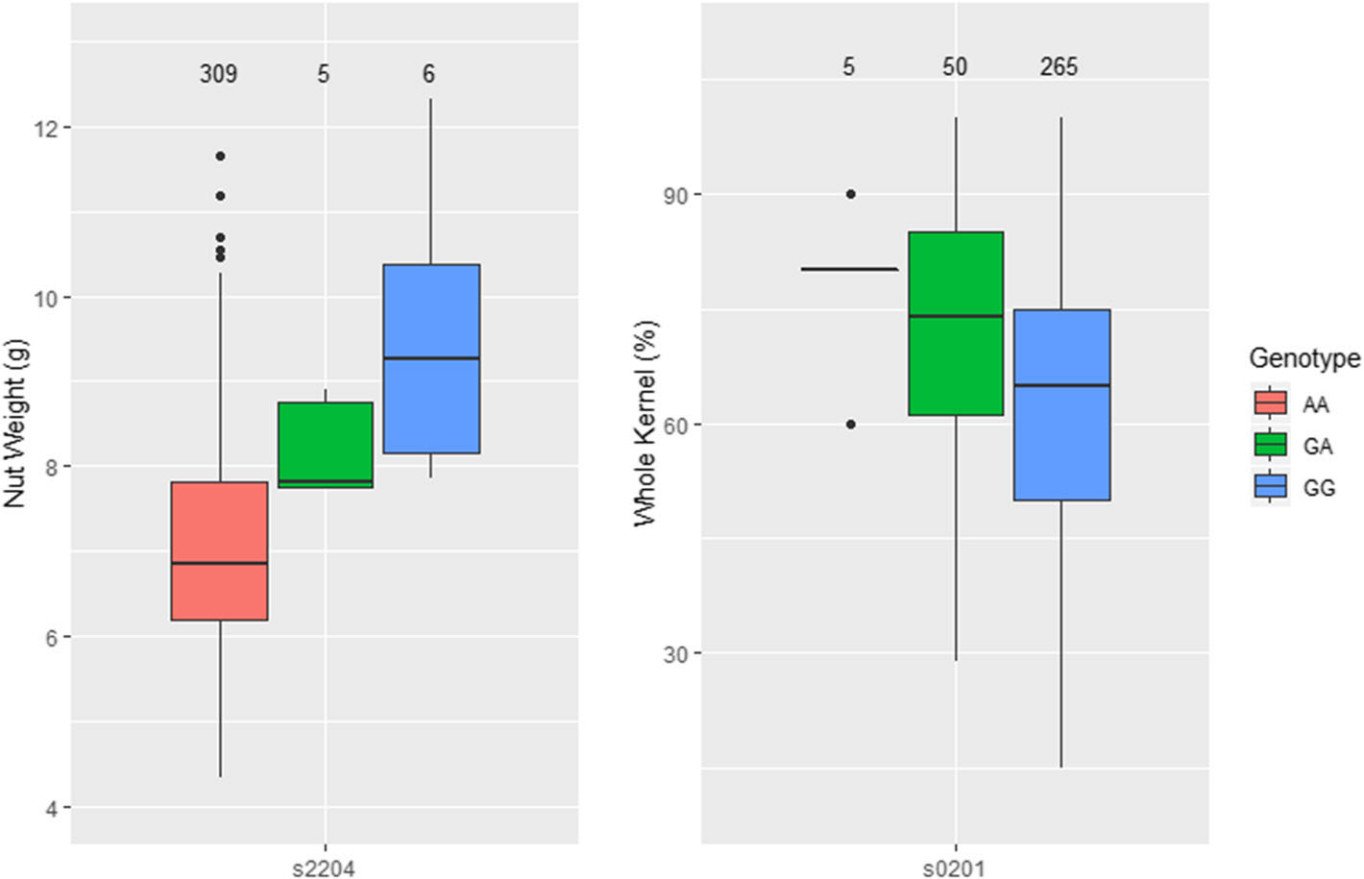
Distribution of raw phenotypes across genotypic states for nut weight and percentage of whole kernels. Numbers above each box represent the number of trees with that genotype for that marker

The phenotypic (raw, untransformed) distributions across the three genotypic states were examined with boxplots for the most significant marker for NW and WK (Fig. 3). The average phenotypes of NW at SNP s2204 for AA, AG and GG genotypes were 7.03 g (*n* = 309, SD = 1.29), 8.20 g (*n* = 5, SD = 0.58), and 9.54 g (*n* = 6, SD = 1.73), respectively (Fig. 3). Similarly, the average values of WK for AA, GA and GG genotypes at marker s0201 were 78.0% (*n* = 5, SD = 11.0), 72.9% (*n* = 50, SD = 15.3), and 62.3% (*n* = 265, SD = 16.8) respectively (Fig. 3). A two-way unbalanced analysis of variance (ANOVA) found that for NW at s2204 there was a signficiant difference between genotypes AA/AG (*p* < 0.05) and AA/GG (*p* < 0.001) but not for AG/GG, and for WK at s0201 a significant difference existed between genotypes AA/GG and AG/GG (*p* < 0.001), but not AA/AG.

## Discussion

### Phenotypic data in the breeding program

Large phenotypic diversity was observed for many of the traits in this study. Average phenotypic values observed here for NW, KW and KR were all slightly higher compared with the same traits in the preliminary study when the trees were young [32]. The moderate heritabilities suggest that selection for a number of traits will result in good genetic progress. For example, the high narrow-sense heritability observed for KR (h^2^ = 0.74) means that the aim to select for higher KR is achievable with truncation selection. This form of selection is where trees with phenotypes or estimated breeding values below a certain threshold are excluded from parent populations, and the mean values of progeny should increase for this trait over generations [34]. Results of this study differed to that in the preliminary study [32] which analysed the same population when the trees were younger (around 8 years of age). Heritability for KR was higher in mature trees than young trees (0.62), whilst KW was lower in mature trees (0.37) than young trees (0.53). In comparison, the difference in heritability for NW between the two studies was low (0.03), but the correlation between these phenotypes was only moderate (0.56).

This study demonstrates that linear mixed models are useful for analysing phenotypic and genetic data in macadamia to identify QTLs for target traits, which is beneficial, as developing new macadamia varieties is time-consuming, laborious and expensive. Additionally, the large tree size and numbers involved in macadamia breeding means that multiple environments are typically needed during evaluation trials. The mixed models employed in this study account for the average effect of the environment, as well as G x E interactions for some traits. Thus, the best model was fitted to the data on a trait-by-trait basis.

### Genetic data

The current study used 4113 SNP markers imputed with high accuracy, though analysis of LD using the same markers and population found that LD declined rapidly over short distances [34]. The number of markers in the current study is comparable with other studies in fruit trees [13, 15–17]; however, the fragmented nature of the macadamia genome scaffolds means the distribution of markers across the whole genome is still unknown. Genetic linkage maps have been used to anchor scaffolds to chromosomes (Langdon et al. *in preparation*), and the location of scaffolds in the genome will be informative for determining locations of genes detected by SNPs in this study.

Population structure affects LD, and this needs to be accounted for in GWAS to avoid spurious associations and over-prediction of allelic effects. For most traits investigated here, the QQ plots showed that only the highly significant markers deviated from the null expectation (y = x line), and did not show inflation of the observed versus expected *p*-values at lower significance levels. QQ plots showing this pattern demonstrate that population structure has been effectively accounted for by the GRM [33]. One explanation for divergence from the null hypothesis (more associations detected than expected) at high p-values is polygenicity: many loci of small effect contributing to variation in the trait [36]. This genetic model may explain the pattern observed for TC, where a large number of associated markers was detected even at low *p*-values. The previous study [32] did not use markers with missing data imputed with high accuracy, and deviations from the null hypothesis line were observed. Imputation of missing data with high accuracy can, therefore, more accurately capture the realised kinship between individuals, and, as such, produce more accurate association results.

### Association analysis

MAS, using the findings of GWAS, is effective for traits controlled by few genes, and, as such, has little value for complex traits like yield [37–39]. However, Kelner et al. [40] performed QTL mapping and found two clusters of QTLs related to fruit yield and cumulative yield in apple on two different linkage groups, as well as QTLs for precocity and biennial bearing. Genomic selection may be a more appropriate and accurate method to predict yield in macadamia [19].

This study identified SNP markers significantly associated with NW, WK and TC. Although no significantly associated markers were detected for KW or KR, the marker with the lowest *p*-value in each case should be investigated in further studies. Neither NPR nor RSN had any significant associations, which may be partly due to the very low heritability of both traits. Additionally, while there was no G x E detected in RSN, there may be a large environmental influence on the capacity of a tree to retain racemes from flowering through to nut set [27, 28].

For TC, 16 of the 44 significant markers were non-redundant, suggesting that there may be 16 QTLs controlling this trait. Multiple regression suggested that all of the the markers significantly associated with NW may have detected the same or linked QTLs, with the most significant SNP (s2204) being the only non-redundant marker. The location of scaffolds in linkage groups (Nock et al. *in preparation*) may further aid the understanding of whether markers are in LD or are separate QTLs.

A direct comparison cannot be made between SNPs found to be significantly associated with nut traits in the preliminary study by O’Connor et al. [32] and the current study, as two different SNP panels were used in the analyses. However, some of the significant markers could be mapped to genome assembly scaffolds. A comparison of the locations of mapped SNPs between the two studies showed that there were no markers occupying the same scaffold (data not shown). Results from GWAS are not always consistent, with variation between populations and environments altering allelic frequencies and phenotypes. For example, differences were found across years in apple [18], and between QTL mapping and GWAS studies in chestnut [11, 41], and this may be a consequence of limited power in these studies.

Researchers use different thresholds for determining which markers to include in their genomics studies, such as 5% MAF [11, 17], 1% MAF within-populations [42], and ten copies of the minor allele across samples [18]. In the present study, markers were initially excluded with MAF < 2.5%, though these statistics were calculated for each marker before imputation, and, as such, the study included markers with MAF below this threshold (MAF altered after imputation of missing calls). It was interesting, then, that all of the markers associated with NW had very low MAF. If these markers had been removed by filtering, they would not have been detected through GWAS. Associations with rare alleles should be treated with caution due to low power of detection [43], and this is the case here. Therefore, the significant markers with low MAF in the current study should be validated in independent studies, preferably with more individuals to observe whether the MAF is similar across populations of different sizes [44], as this will support the findings of this study.

### Demonstration of marker-assisted selection

The results of this GWAS study can be used to demonstrate the implementation of MAS in the macadamia breeding program. SNPs significantly associated with commercially important traits would be ideal candidates for use in MAS. The estimates of BLUEs in the multiple regression analysis indicate the additive effect of the SNP allele at that marker on that trait. For example, the estimated effect for SNP allele at s2204 was 0.084, meaning that genotypes with one SNP allele will have an added 0.084 g of nut weight controlled by genetic variance than those without. The influence of additive genetic variance of these alleles was quite different to that which was observed in the raw phenotype, as the phenotype will have been influenced by non-additive genetic effects and environment. The three genotypic states for NW at SNP s2204 and for WK at marker s0201 showed clear association with phenotypic averages, though the difference in genotypic states was much lower than the additive allele effect calculated from BLUEs. The sample sizes among the three different genotypic states varied greatly in these examples, and so it is important to recognise that these findings are severely biased upwards and are only for demonstrative purposes for how MAS could be used. Simply, breeders could genotype seedling progeny from their first leaf at these key markers. Determining the allelic states at these markers would allow selection of AG heterozygotes at SNP s2204 for seedlings with predicted intermediate nuts, and AA genotype at SNP s0201 for a high percentage of whole kernels. However, with such low MAF and number of individuals in these genotypic states, these results should be interpreted with caution. Again, the SNP should be validated in an independent population, and the effect of the SNP alleles should be estimated in that population.

### Further work

This study and our previous work [32] provide a foundation for how the use of genomics can improve breeding in macadamia, and is among the first to analyse the potential for genomics-assisted breeding in nut crops. However, the results presented require validation before being employed in breeding programs. Multi-trait analyses could be performed to increase the power of detection of QTLs, and also detect pleiotropy [45]. A separate population should be studied to determine if QTLs detected are the same as those detected here, or are new associations. Further studies should incorporate larger population sizes, to ensure that significant associations are accurate and applicable to a wider breeding population. Additionally, the low MAF observed for some markers in this study may change with sample size, which will influence the proportion of variance explained by those markers.

When a more complete reference genome is assembled, the location of these markers can be determined, and LD between markers more accurately estimated with population structure and cryptic relatedness taken into account. Due to the rapid decay of LD over short distances in macadamia [34], using a larger number of markers may increase the likelihood of SNPs being in LD with causal polymorphisms. Furthermore, the potential issues posed by allelic dropouts, such as lower than expected levels of heterozygosity observed by O’Connor et al. [34], could be alleviated with the use of a complete reference genome in sequencing of SNPs in the future. Without genome scaffold annotation, the significant SNPs cannot be linked to known genes or proteins, which has been achieved in other studies of GWAS in fruit trees (e.g. [13, 15, 16, 18]). The v2 scaffolds and chromosomes are being (Nock et al. *in preparation*), and so candidate genes could be identified in future studies.

Although there was a lack of significant associations in some traits in the current study, these should still be investigated in future work. The polygenic nature of TC, as well as the complexity of yield, means that these traits may be more suited for genomic selection, where many markers may have a small effect on the trait, and all markers are modelled simultaneously [46], rather than one-by-one as in GWAS. Other traits that could be analysed include self-fertility, and resistance to diseases that affect nut yield, including husk spot and phytophthora.

## Conclusions

The findings of this study have important implications for macadamia breeding, but also highlight the difficulties of employing GWAS in heterozygous populations with rapid LD decay. Significant associations were detected for NW, WK and TC, but no markers exceeded the significance threshold for KW, KR, RSN or NPR. The traits with significant SNPs identified are likely to be controlled by fewer genes than the other traits. Multiple regression determined that several significant markers were detecting the same QTL, and, as such, were redundant. By coupling validated marker-trait associations detected through GWAS with MAS, genetic gain could be increased by reducing the selection time for economically important nut characteristics and other yield component traits. Genomic selection may be a more appropriate method to predict complex traits like yield. This study provides a foundation for genomics-assisted breeding in macadamia and nut crops more broadly, and advances our understanding of the genetic control of yield component traits.

## Methods

Methods for association analysis are similar to those in a preliminary study by O’Connor et al. [32], and are replicated here for completeness, with differences between the two studies outlined.

### Study design

This study involved 295 seedling progeny from 32 full-sib families, as well 18 of their 29 parents (that were phenotyped), from the Australian macadamia breeding population. Trees were planted between 2001 and 2003 across four sites in Queensland, with East Gympie (EG) and Amamoor (AM) in the Gympie region, and Alloway (AL) and Hinkler Park (HP) in the Bundaberg region. Clones of five of the parents were measured at all four sites. Yield and yield component traits were measured on each tree between 2016 and 2018; hence, trees were mature-aged (aged 14–17 years). Details of genotyping methods for this population were reported in O’Connor et al. [34], which used the same individuals and SNP markers. Briefly, leaf samples from each genotype were sequenced by Diversity Arrays Technology (DArT) Pty Ltd. SNP markers were imputed by DArT, with 97.2% accuracy using the probabilistic principal components analysis method [47]. Markers were filtered for various quality control measures (based on pre-imputation genotypes), and those that passed thresholds were retained for analysis. The quality control measures included > 50% call rate, > 2.5% minor allele frequency, > 0 polymorphic information content, and a test of Mendelian consistency between progeny-parent-parent trios in half of the studied families. This gave 4113 SNP markers for analysis.

### Phenotyping for yield and component traits

Phenotypic data used in this study were collected across two seasons from August 2016 to July 2018, with all traits except RSN and yield measured only in one season. A sample of nuts was taken from each tree and dried to 1% moisture content in an oven at 35 °C for 2 days, 45 °C for 2 days and 55 °C for a final 2 days, based on protocol by Prichavudhi and Yamamoto [48]. Twenty good quality nuts (no kernel shrivelling or pest damage) were chosen to measure four traits. Nuts were individually weighed to obtain nut weight (NW). Nuts were then manually cracked, and kernel and shell separated to record kernel weight (KW). Kernel recovery (KR) was calculated as KW / NW. The percentage of whole kernels (WK) per sample was measured as the proportion of nuts that did not split between the two cotyledons during cracking.

Tree trunk circumference (TC) was measured at a height of 50 cm above the ground, or below any low branches. Flowering racemes present in a 30 cm length of branch, 20 cm from the branch apex were flagged and counted on two branches per tree. Where necessary, trees with terminal racemes were also flagged and counted, to make a total of at least ten racemes per tree. At nut maturity (around March, Australian autumn), the number of flagged racemes that had set at least one nut was counted, and the percentage of racemes that survived from flowering through to nut set (RSN) was calculated. The number of nuts per raceme (NPR) was counted from ten racemes per tree. Component trait means were calculated for each tree for analysis where at least six observed units per tree were evaluated. For example, trees with five or fewer nuts measured were considered to have missing data for this trait. Mean RSN was calculated for each tree over the 2 years.

Yield data were collected from March through to July over two successive seasons in multiple harvests. Yield was measured on each tree by manually harvesting nuts from the ground and collecting any nuts still in the tree at the end of the season. Nuts were dehusked after each harvest, weighed, and a 1 kg sample was dried to 1% moisture content. The dry nut-in-shell (DNIS) weight was estimated for each harvest using calculations of moisture content in the 1 kg sample. The DNIS weight for each harvest was summed across the whole season to give total DNIS yield. One site (AL) was not harvested in 2017 due to an extreme weather event, and in 2018 another site (EG) was not harvested due to management issues.

Histograms were used to check the distribution of phenotypes to conform with assumptions of normality for GWAS [43]. Data transformations were performed where necessary to normalise distributions. Pearson’s correlations were performed between NW, KW and KR raw phenotypes in the current study and those used in O’Connor et al. [32] to investigate the consistency of phenotypes between the two studies.

### Association analysis

A genomic relationship matrix (GRM) was constructed following methods of VanRaden [49]. Preliminary analysis was performed using ASReml [50] in R to determine the most parsimonious model for each trait:

1 $$ \mathbf{y}=\mathbf{1}\boldsymbol{\upmu } +\mathbf{Xb}+{\mathbf{Z}}_{\mathbf{g}}\mathbf{g}+{\mathbf{Z}}_{\mathbf{g}\mathbf{s}}\mathbf{g}\mathbf{s}+\mathbf{e} $$

where **y** is a vector of phenotypes, **1** is a vector of ones, **μ** is a fixed intercept, **X** is a design matrix allocating fixed effects (site, block within site, tree type = grafted parent or seedling progeny) to observations, **b** is a vector of these unknown fixed effects, **Z**_**g**_ is a design matrix allocating records to the unknown average breeding value of each individual across sites; **g** is a vector of averaged breeding values of the individuals across sites, assumed random ~ N (0,**G** ${\sigma}_g^2$), where **G** is the additive genomic relationship matrix (GRM) among the individuals, modelled from SNP effects (0, 1, and 2 represent homozygous, heterozygous and alternate homozygous genotypes, respectively); ${\sigma}_g^2$ is the genetic variance captured by the SNP; **Z**_**gs**_**gs** describes the genotype by environment (G x E) interaction, where **Z**_**gs**_ is a design matrix allocating a specific effect of an individual at a site not accounted for by the mean of the individual across sites, and **gs** is a vector of the breeding values at a specific site, assumed random ~ N (0,**G**⨂**I**_**4**_⨂ ${\sigma}_{gs}^2$) where **I** is a 4 × 4 identity matrix for the four sites, and **e** is a vector of random errors ~ N (0, ${\sigma}_e^2$) where ${\sigma}_e^2$ is the error variance. This model is additive, in that two copies of one allele will have double the effect of one copy.

Preliminary analyses determined the significance of fixed effects site, block within site, and tree type (grafted parent or seedling progeny) using the Wald statistic. After removing insignificant fixed effects (individualised for each trait), log likelihoods of models both including and excluding G x E as a random term were compared via a chi-square test to determine if the models were statistically different. The most parsimonious models were those with the least number of parameters that fit the data as well as more complex models: the G x E term was excluded for a trait if the models were not statistically different, as well as any insignificant fixed effects. Narrow-sense heritability (h^2^) was calculated from variance components (h^2^ = ${\sigma}_g^2$ / (${\sigma}_g^2$ + ${\sigma}_e^2$)) for each trait using the best-fitting model. For traits where G x E was a significant factor, the G x E variance component was included in the denominator when calculating heritability.

Association analysis was performed for each trait using the most parsimonious model, as per O’Connor et al. [32] using ASReml [50] in R, using a mixed model:

2 $$ \mathbf{y}=\mathbf{Xb}+\mathbf{Wm}+{\mathbf{Z}}_{\mathbf{g}}\mathbf{g}+{\mathbf{Z}}_{\mathbf{g}\mathbf{s}}\mathbf{g}\mathbf{s}+\mathbf{e} $$

where **W** is a design matrix allocating records to the marker effect (modelled as 0, 1, or 2 for homozygous, heterozygous and alternate homozygous genotypes, respectively), and **m** is the effect of the marker currently being fitted in the model, as a fixed effect. All other effects are the same as per Eq. 1.

QQ (quantile-quantile) plots were constructed for each trait to evaluate whether population structure had been accurately accounted for in the model, by comparing the observed and expected –log_10_ significance values of each SNP and ensuring that inflation had not occurred at the lower levels of significance [43]. To determine a threshold above which markers were deemed significantly associated with a trait, a false discovery rate (FDR) was calculated for each trait with the BH method [51] using the p.adjust function in R. Markers with FDR < 0.05 were deemed significantly associated with the trait. Multiple regression was performed for traits with multiple significant associations based on the best-fit model, where significant markers were included as fixed effects, to determine if any SNPs were in LD. FDR was again calculated for the markers included in the multiple regression. Markers that were no longer significant after multiple regression were deemed to be detecting the same QTL as one of the significant markers, and as such were considered redundant. An estimation of the additive allele effect of each significant SNP was estimated from fixed effects (best linear unbiased estimators; BLUEs) from the multiple regression model.

### Marker locations

Locations of significant SNPs (FDR < 0.05) on the most recent macadamia genome scaffolds (v2; 4098 scaffolds; European Nucleotide Archive (EMBL-ENA) repository, Analysis: ERZ792049, Assembly accession: ERS2953073 (SAMEA5145324)), were estimated as per O’Connor et al. [34]. Locations of previously identified markers associated with nut traits were also estimated on the scaffolds, using marker sequences from the preliminary study [32].

## Data Availability

The data that support the findings of this study are available from The University of Queensland’s Institutional Data Access/Ethics Committee, but restrictions apply to the availability of these data. Data are however available from the authors upon reasonable request and with permission of The University of Queensland for researchers who meet the criteria for access to confidential data. Contact data@library.uq.edu.au.

## Abbreviations

DArT: Diversity Arrays Technology
DNIS: Dry nut-in-shell
FDR: False discovery rate
G x E: Genotype by environment interaction
GRM: Genomic relationship matrix
GWAS: Genome-wide association study
KR: Kernel recovery
KW: Kernel weight
LD: Linkage disequilibrium
MAF: Minor allele frequency
MAS: Marker-assisted selection
NPR: Number of nuts per rachis
NW: Nut weight
QQ: Quantile-quantile
QTL: Quantitative trait loci
RSN: Racemes surviving from flowering to nut set
SNP: Single nucleotide polymorphism
TC: Trunk circumference
WK: Whole kernels
